# Clinical Usefulness of a Short Version of the Internet Addiction Test to Screen for Probable Internet Addiction in Adolescents with Autism Spectrum Disorder

**DOI:** 10.3390/ijerph20054670

**Published:** 2023-03-06

**Authors:** Masaru Tateno, Kazumasa Horie, Tomohiro Shirasaka, Kotaro Nanba, Eri Shiraishi, Yukie Tateno, Takahiro A. Kato

**Affiliations:** 1 Tokiwa Child Development Center, Tokiwa Hospital, Tokiwa 3-1-6-1, Minami-ku, Sapporo 0050853, Japan; 2 Department of Neuropsychiatry, Graduate School of Medicine, Sapporo Medical University, South-1, West-16, Chuo-ku, Sapporo 0608543, Japan; 3 Department of Neuropsychiatry, Graduate School of Medical Sciences, Kyushu University, Maidashi 3-1-1, Higashi-ku, Fukuoka 8128582, Japan; 4 Department of Psychiatry, Teine Keijinkai Medical Center, Maeda 1-12-1-40, Teine-ku, Sapporo 0060811, Japan

**Keywords:** internet addiction, problematic internet use, gaming disorder, behavioral addiction, internet addiction test, autism spectrum disorder, attention deficit hyperactivity disorder, neurodevelopmental disorders

## Abstract

Internet addiction (IA) is defined as the condition of being addicted to all sorts of activities on the Internet. Individuals with neurodevelopmental disorders, including autism spectrum disorder (ASD), may be susceptible to IA. Early detection and intervention for probable IA are important to prevent severe IA. In this study, we investigated the clinical usefulness of a short version of the Internet Addiction Test (s-IAT) for the screening of IA among autistic adolescents. The subjects were 104 adolescents with a confirmed diagnosis of ASD. They were requested to answer 20 questions from the original Internet Addiction Test (IAT). In the data analysis process, we comparatively calculated the sum of scores to the 12 questions of s-IAT. In total, 14 of the 104 subjects were diagnosed as having IA based on the face-to-face clinical interview that was regarded as the gold standard. Statistical analysis suggested that the optimal cut-off for s-IAT was at 35. When we applied the cut-off of 70 on the IAT, only 2 of 14 subjects (14.3%) with IA were screened positive, whereas 10 (71.4%) of them were screened by using the cut-off point of 35 on s-IAT. The s-IAT might be useful for the screening of IA in adolescents with ASD.

## 1. Introduction

The Internet has made our lives very convenient and has become an indispensable tool for our daily lives. However, in recent years, various problems related to excessive internet use are attracting social interest. Internet addiction (IA), otherwise referred to as problematic internet use (PIU), is defined as the condition of being addicted to all sorts of activities on the Internet, including online gaming, video sharing, listening to music, and social media usage [1]. As the number of Internet users becomes higher, problems related to internet use become more serious, especially among adolescents [2].

Previous studies have demonstrated that neurodevelopmental disorders, especially attention deficit hyperactivity disorder (ADHD), are related to IA [3,4,5]. A comparative meta-analysis of neuroimaging studies has suggested that internet gaming disorder (IGD) and ADHD might have shared and special structural and functional alterations in common [6]. A 2-year prospective study which invited more than 2000 junior high school students has revealed ADHD to be the most significant predictor for the development of IA [7]. The results of our survey reported that ADHD was a common comorbidity of IA from the perspective of child and adolescent psychiatrists [8]. Almost all studies which investigated the possible relationship between IA and those individuals with ADHD demonstrated positive findings [9].

However, results from studies about IA among subjects with ASD were somewhat inconsistent [10]. Several studies have reported that individuals with ASD had a higher prevalence of IA compared with those without ASD [3,4]. In contrast, some studies failed to conclusively demonstrate that subjects with ASD were more susceptible to IA [11]. Even in nonclinical samples, a study conducted by Chen et al. among junior high school students and their parents failed to demonstrate a positive relationship between ASD traits and IA [12]. One possible explanation of this difference might be related to the nature of behavioral addiction that largely relies on self-reports of clinical symptoms. Many previous studies on IA have measured the severity of addiction using a self-reporting scale. Subjects with ASD may have difficulties in assessing their own inappropriate behaviors objectively [13] or have poor insight into their own behavioral problems because of deficits in social skills [14]; therefore, they may be prone to underestimating their problematic issues related to the Internet compared with neurotypical individuals. Above all, regarding studies of IA, very few studies have conducted face-to-face interviews, and many studies are based solely on the results of self-rating scales.

Reflecting a significant concern about IA all over the globe, several self-rating scales to screen IA have been developed to date [15], such as the Compulsive Internet Use Scale (CIUS) [16], the Chen Internet Addiction Scale (CIAS) [17], Young’s Diagnostic Questionnaire for Internet addiction (YDQ) [18], and the Internet Addiction Test (IAT) [19]. Among them, the most frequently used scale to screen IA worldwide is definitely the IAT, which has been translated into many other languages and used in several countries, e.g., France [20], Germany [21], Spain [22], Italy [23], Greece [24], Turkey [25], Croatia [26], China [27], South Korea [28], Thailand [29], Indonesia [30], and Japan [31].

The IAT consists of 20 questions regarding frequency of problems related to internet overuse on 5-point Likert scale. The questions of IAT comprise several facets of behavioral addiction, including preoccupation, loss of control, and psychological dependence. However, the factorial structure of the IAT is still controversial [32,33,34]. The number of factors extracted from the IAT reported in previous studies varies from one to two, three, and four factors. These inconsistent results are likely to depend on the methodology of each study, such as sample size, the methods of factor analyses, and characteristics of study participants [33]. It has also been more than two decades since the IAT was developed by Young, referring to diagnostic criteria for pathological gambling in the Diagnostic and Statistical Manual of Mental Disorders 4th ed. (DSM-IV) [19,35]. In that time, the information and communication technology surrounding us has changed dramatically. For example, one of the items of the IAT is “How often do you check your e-mails before something else that you need to do?” Nowadays, for the young generation, the most common tools to communicate with peers are social networking services (SNSs), e.g., WhatsApp, Instagram, Twitter, and Facebook. In Japan, a free application named LINE, which is mainly designed for text chatting on smartphones, is the most popular social media platform among young people [36]. Furthermore, this item asks about Internet use for a specific purpose, while many other items are questions related to Internet use in general [15]. To solve these issues, Pawlikowski et al. proposed a short version of the IAT (s-IAT) [33] consisting of 12 items extracted from the original IAT after a thorough investigation of the results of four large-scale studies that invited different subject groups for each study.

In this study, we investigated the clinical usefulness of s-IAT for the screening of IA among autistic adolescents.

## 2. Materials and Methods

### 2.1. Study Subjects

This study was performed as a single-site survey, and subjects were recruited at the outpatient clinic of Tokiwa Child Development Center in Sapporo, Japan. The locus of this study, Sapporo, is a major metropolitan area in northern Japan with a population of approximately 1.95 million. All eligible adolescents with a confirmed diagnosis of ASD in an age range of 10 to 18 years old were invited to participate in the study, except for those with moderate to severe intellectual disabilities, referring to the results of Wechsler Intelligence Scale tests that all of them had undergone in addition to a thorough clinical assessment performed by experienced clinicians. The clinical diagnosis of ASD was confirmed by a consensus among a multi-professional team including board-certified child psychiatrists, as per the Diagnostic and Statistical Manual of Mental Disorders, Fifth Edition (DSM-5) [35]. All subjects had visited our clinic on a regular basis for at least 6 months prior to the data collection; this fact suggests the relatively higher reliability of the clinical diagnosis of ASD.

### 2.2. Internet Addiction Test (IAT)

The IAT was developed by Young as a diagnostic instrument for IA on the basis of the DSM-IV criteria for pathological gambling [19]. The IAT consists of 20 questions regarding the frequency of internet use. All questions begin with a sentence such as ‘How often do you…?’, for example, or ‘How often do you find that you stay online longer than you intended? (Q1)’ Response choices range from 1 = rarely to 5 = always. The original cut-off points of IA are 70 and higher, although several studies recommend 50 and higher for the reasonable cut-off [31,37]. A Canadian research group has demonstrated that a cut-off of 50 could be appropriate to screen subjects with probable Internet use among 3938 healthy adolescents [37]. The reliability and validity of the Japanese version of the IAT has been investigated [38,39].

### 2.3. A Short Version of the Internet Addiction Test (s-IAT)

A short version of the IAT consists of 12 questions selected from the 20 questions in the original IAT with a two-factor structure: loss of control/time management and craving/social problems [33]. The s-IAT was developed not only to shorten the original IAT, but also to consider the dramatic changes in the Internet environment surrounding us. The IAT was developed more than 20 years ago; thus, it contains questions that are not in line with how contemporary teenagers use the Internet. One example is checking e-mails by desktop or laptop computers, because today, teenagers will check their messages using other electronic devices such as smartphone. In addition, the IAT contains similar questions, and the repetition of questions may lead to overestimations of certain behaviors. The s-IAT has been translated into several languages, such as French [40], Spanish [41,42], German [43], and Vietnamese [44]. The usefulness of the s-IAT has been reported by these studies, and a modified version of s-IAT has previously been used for specific purposes, such as Internet communication disorder [43], online sexual activities [40], social networking sites [41,42], and gaming [15].

The psychometric properties and adequate reliability of the s-IAT have been demonstrated. The s-IAT has just 12 items; therefore, the total score ranges from 12 to 60, and the cut-off points of IA has been proposed as 37 by the developer. The cut-off of 37 was set based on the results of a sample of 465 study subjects, consisting mostly of college students in Germany.

In this study, we asked all subjects to answer 20 questions of the original IAT and, in the process of data analyses, we comparatively calculated the sum of scores to the 12 questions of the s-IAT.

### 2.4. Clinical Diagnosis of IA

To date, there are no established diagnostic criteria of IA. Thus, in this study, we performed a clinical diagnosis of IA according to the description of gaming disorder (GD) of the International Classification of Diseases 11th Revision (ICD-11) [45]: (1) impaired control over the use of the Internet (e.g., onset, frequency, intensity, duration, termination, and context); (2) increasing priority given to online activities to the extent that Internet use takes precedence over other life interests and daily activities; and (3) escalation of Internet use despite the occurrence of negative consequences. The pattern of Internet overuse may be continuous or episodic and recurrent. The pattern of Internet overuse results in marked distress or significant impairment in personal, family, social, educational, occupational, or other important areas of functioning. The clinical diagnosis of IA was confirmed in reference to additional thorough information provided by the subjects’ parent(s). To maximize the inter-rater reliability regarding the clinical diagnosis of IA, a semi-structured interview form for the clinical diagnosis of GD developed by Higuchi et al. was used in this study [46]. The semi-structured interview form is provided as Supplementary Material in their paper on a GAMES test that was developed as a screening tool for GD [46].

### 2.5. Statistical Analysis

We performed Welch’s *t*-test to compare the mean of two groups and calculate sensitivity- and specificity-related factors using StatFlex Ver.6 (Artec Inc., Osaka, Japan). We also used R version 4.1.2 (R Foundation for Statistical Computing, Vienna, Austria) for other statistical analyses [47]. The exact 2 × 2 package [48] was used for McNemar’s test, while the pROC package [49] was used for receiver operating characteristic (ROC) analysis. Some cells had a small number of samples; therefore, Fisher’s exact test in the stats package was used for statistical analyses [47]. Statistical significance was set at *p* < 0.05.

### 2.6. Ethics

The study protocol was approved by the ethics committee of Tokiwa Hospital (TH-180219). This study was performed in accordance with the Declaration of Helsinki. Informed assent and informed consent were obtained from the subjects and their guardians, respectively.

## 3. Results

### 3.1. Sociodemographic of the Subjects

In total, 104 patients were invited to participate in this study. Sociodemographic information of the subjects is summarized in Table 1. More detailed results on age, gender, the presence and absence of IA, and total scores of the IAT and s-IAT are presented in Table S1 in the Supplementary Material. The mean age of the subjects was 14.4 ± 2.4 years. The mean total scores of the IAT and s-IAT were 43.7 ± 12.4 and 27.4 ± 7.9, respectively. Regarding the age category of the subjects, there were 57 aged 10–14 years old (54.8%) and 47 aged 15–18 years old (45.2%). The number of subjects who scored more highly than each cut-off is shown in Table 2. Only two of the subjects were screened positive by IAT with the cut-off as 70, whereas 10 were screened positive with the cut-off of 50.

### 3.2. Clinical Diagnosis of IA

Regarding the clinical diagnosis based on the repetitive face-to-face examinations, 14 (13.5%) of the 104 subjects were diagnosed as having IA with the modified application of the diagnostic guidelines for GD of ICD-11. In this study, the clinical diagnosis was regarded as the gold standard.

Two group comparisons using Welch’s *t*-test between non-addicted and addicted respondents revealed that there were no significant differences in age and intelligence quotient (IQ) as measured by Wechsler Intelligence Scale tests of the subjects, as shown in Table 1. On the other hand, the total scores of both IAT and s-IAT were higher in addicted subjects.

### 3.3. Cut-Off Points for IAT and s-IAT

The appropriate cut-off points for the 20-item original IAT and s-IAT in the present subjects were investigated based on the clinical diagnosis according to face-to-face examinations. Receiver operating characteristic (ROC) analyses were performed to assess the optimal cut-off points for both scales.

**Figure 1:**
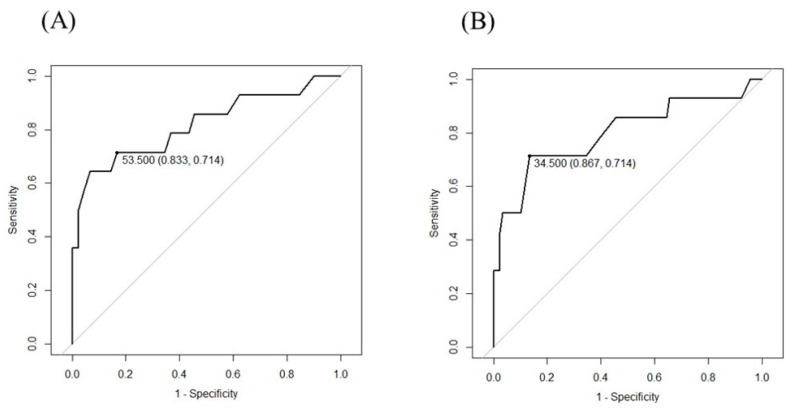
Receiver operating characteristic (ROC) analysis for the IAT (**A**) and the s-IAT (**B**). ROC: receiver operating characteristic; IA: internet addiction; IAT: Internet Addiction Test; s-IAT: the short version of the Internet Addiction Test.

Regarding the 20-item IAT, the Cronbach’s alpha value for internal consistency was 0.8587, and the area under the curve (AUC) was 0.8171 (95% CI: 0.6684–0.9657). Statistical analysis based on the point on the ROC curve closest to (0, 1) suggested that the total score of 53.5 could be the optimal cut-off to screen IA, as shown in Figure 1A. For the IAT, because scores of 70 or 50 have been widely used as the consensus cut-off points for IA, we adopted the total score of 50 in this study.

Similarly, with regard to the s-IAT, a Cronbach’s alpha value of 0.7883 confirmed the valid internal consistency of this scale. AUC was 0.7992 (95% CI: 0.6432–0.9552). The optimal cut-off for the s-IAT was suggested as 34.5, based on the Youden index, as shown in Figure 1B. Thus, in this study, we set the cut-off point as 35 in total for s-IAT. This cut-off point was the same as the cutoff proposed in a study by Müller et al., whose subjects were 18–31-year-olds [43].

Cronbach’s alpha values of the IAT for the two groups divided by age (early teens, 10–14 y; late teens, 15–18 y) were 0.8456 and 0.8750, respectively. Similarly, Cronbach’s alpha vales of the s-IAT for the two groups divided by age (early/late teens) were 0.7657 and 0.8129, respectively.

Statistical analysis based on the point on the ROC curve closest to (0, 1) suggested that the total score of 53.5 on IAT could be the optimal cut-off to screen IA, as shown in Figure 1A, and 34.5 on the s-IAT based on the Youden index, as shown in Figure 1B.

### 3.4. Results of the IAT and s-IAT for Screening IA

The results are summarized in Table 2. When we applied the cut-off of 70 and higher in total, only 2 (14.3%) of 14 subjects diagnosed as IA by face-to-face examinations were screened positive, while 10 (71.4%) of them were screened positive by using the cut-off points of 50 and higher on 20-item conventional IAT. Regarding the s-IAT, 10 (71.4%) of 14 subjects with a clinical diagnosis of IA scored 35 and higher and were screened positive. Regarding the presence and the absence of probable IA and the number of the subjects whose score exceeded cut-off points, Fisher’s exact test demonstrated that there was no significant difference in the number of subjects who scored more highly than each cut-off. The sensitivity, specificity, false positive rate (FPR), false negative rate (FNR), positive predictive value (PPV), negative predictive value (NPV), positive likelihood ratio (PLR), and negative likelihood ratio (NLR) were calculated for each cut-off for either the s-IAT or the IAT, as summarized in Table 3. McNemar’s test for the s-IAT and IAT at two different cut-off points (CO) showed significant distribution bias in the s-IAT (CO35) and the IAT (CO50), and in the s-IAT (CO35) and the IAT (CO70), as shown in Table 4.

## 4. Discussion

In this study, we investigated the clinical usefulness of the s-IAT in screening for IA in adolescents with ASD. The AUC values of the ROC analysis suggested that the s-IAT had moderate discrimination accuracy that was equivalent to the original IAT. When we used the s-IAT for the screening of IA among adolescents with ASD, it was suggested that applying a cut-off of 35 on the s-IAT as opposed to the conventional cut-off of 37 was better to increase the reliability of the s-IAT. By adjusting the cut-off, the sensitivity, which is important for screening, was similar between the s-IAT and IAT (CO50). The results suggested that the s-IAT is better than the IAT (CO50) regarding the specificity, PPV, NPV, FPR, PLR, and NLR to screen for IA among adolescents with ASD. On the other hand, the sensitivity of the IAT (CO70) is quite low and might not be suitable for screening IA in ASD adolescents. Furthermore, considering the results of McNemar’s test and its sensitivity and specificity, it seems that the s-IAT (CO35) may be better than the IAT (CO50) in excluding non-dependent subjects. In addition, the s-IAT (CO35) could screen more probable IA than the IAT (CO70).

Our results demonstrated that the short version of the IAT, the s-IAT, is as useful as the 20-item conventional version to screen ASD adolescents with IA. By asking fewer questions on self-rating scales, we can reduce the burden on respondents. Adolescents with neurodevelopmental disorders often have difficulties in concentrating on any activities due to ADHD symptoms; thus, self-rating scales that can be answered in a short period of time could be helpful and might increase the accuracy.

In our clinical practice as well, it is not uncommon to see individuals with ASD who spend a significant amount of time on the Internet, but whose total scores of IAT remain within the range of normal users, i.e., less than 40. Regarding IA in those with neurodevelopmental disorders, many previous studies have consistently reported a significant relationship between ADHD and IA, although the results for ASD have been inconsistent. There seem to be several possible reasons to explain this finding.

One possible reason might be that the s-IAT excludes several questions which are based on the key premise of measuring the extent of interaction with others, e.g., “Do you check your e-mail before anything else that you need to do?” According to the diagnostic criteria in DSM-5, ASD is characterized by the following two dimensions: (a) persistent deficits in social communication and social interaction across multiple contexts; and (b) restricted, repetitive patterns of behavior, interests, or activities. Some of the questions of the IAT that require reciprocal interaction with others could be scored lower in those with ASD.

Difficulties in developing good relationships and friendship with peers due to deficits in social interaction abilities could potentially induce even greater social isolation and might increase the time spent on the Internet. Some individuals with ASD prefer to experience online interactions with others while hiding their real selves, and act out a personality (represented by an ‘avatar’) that is quite different from their true character. In this study, we did not ask the purpose of their Internet use. However, a national survey on Internet use in Japan has reported that most young male teens mainly use the Internet for gaming [50]. In the first author’s clinical experience, many male gamers play online games using an avatar, and although they may be calm and modest in their real lives, they are often quite active and sometimes even very aggressive in the virtual space of gaming. A group of ASD subjects might use the Internet for quite a long time to obtain information about their limited interests. However, the characteristic of repetitive patterns of behavior might work as a protective factor against excessive use of the Internet because ASD individuals with repetitive behavior patterns may want to go to bed at exactly the same time every day and prefer a scheduled pattern of daily life with fixed time slots for designated activities. Some ASD subjects exhibit obsessive behaviors with respect to obeying and/or strictly following either external or self-imposed rules. In such cases, the total score of the IAT will be lower than the cut-off.

Additionally, above all, in reflecting their deficits in social skills, ASD individuals may not be good at objective self-evaluation. Even if they have significant problems related to Internet overuse, some adolescents with ASD may not be aware of them. Needless to say, no psychiatric disorders should be diagnosed by solely relying on the results of self-rating scales. We need to remain cognizant that the scores of self-rating scales by ASD subjects might have limited reliability.

This study has several limitations. The sample size of this study was limited, although all of them underwent face-to-face interviews for the diagnosis of IA. The subjects were a heterogeneous group in terms of age. The study design was cross-sectional. We recruited study subjects only from one institute. The test–retest reliability has not been investigated in this study.

## 5. Conclusions

Our results demonstrated that the s-IAT might be useful for the screening of IA in adolescents with ASD because it may provide an early opportunity to identify incipient disease. Further studies with a larger sample size are warranted to investigate the usefulness of the s-IAT as a reliable screening tool of probable IA in clinical settings.

## Figures and Tables

**Table 1:** Sociodemographic information and scores of the IAT/s-IAT of the subjects.

	Overall	Non-Addicted	Addicted	*p*-Value
	*n* = 104	*n* = 90	*n* = 14	(Welch’s *t*-Test or ^$^ Fisher’s Exact Test)
Age (Age range) Early/Late teens	14.4 ± 2.4 (10–18) 57/47	14.4 ± 2.5 (10–18) 47/43	14.1 ± 1.9 (11–18) 10/4	*p* = 0.5446 *p* = 0.2507 ^$^
Gender (M/F)	80/24	71/19	9/5	*p* = 0.3042 ^$^
IQ	82.8 ± 14.0	82.8 ± 14.1	82.2 ± 14.0	*p* = 0.8798
IAT (20 items)	43.7 ± 12.4	41.6 ± 11.0	56.8 ± 13.1	*p* = 0.0012
s-IAT (12 items)	27.4 ± 7.9	26.2 ± 6.9	35.5 ± 9.0	*p* = 0.0023

The results are expressed as the mean ± standard deviation (SD). Welch’s *t*-test was used to compare the means of two groups. ^$^ Fisher’s exact test was used to compare the male and female distributions. Early teens: 10–14 year old, late teens: 15–18 years old, IQ: intelligence quotient; IAT: Internet Addiction Test; s-IAT: the short version of the Internet Addiction Test.

**Table 2:** The number of the subjects who scored more highly than each cut-off in non-addicted and addicted groups.

	Overall	Non-Addicted	Addicted	*p*-Value
	*n* = 104	*n* = 90	*n* = 14	(Fisher’s Exact Test)
s-IAT ≥ 35	22	12	10	*p* = 0.07986
IAT ≥ 50	35	25	10	
IAT ≥ 70	2	0	2	

IAT: Internet Addiction Test; s-IAT: the short version of the Internet Addiction Test.

**Table 3:** Diagnostic characteristics of the s-IAT and the IAT at different cut-off scores.

	Sensitivity	Specificity	FPR	FNR	PPV	NPV	PLR	NLR
s-IAT (CO35)	0.714	0.867	0.133	0.286	0.455	0.951	5.357	0.330
IAT (CO50)	0.714	0.722	0.278	0.286	0.286	0.942	2.571	0.396
IAT (CO70)	0.143	1.00	0	0.857	1.00	0.882	-	0.857

FPR: false positive rate; FNR: false negative rate; PPV: positive predictive value; NPV: negative predictive value; PLR: positive likelihood ratio; NLR: negative likelihood ratio; IAT: Internet Addiction Test; s-IAT: the short version of the Internet Addiction Test, CO: cut-off points.

**Table 4:** Cross-tabulation table of the s-IAT and IAT at different cut-off scores.

(**A**) s-IAT (CO35) and IAT (CO50)				
	**s-IAT ≥ 35**	**s-IAT < 35**	**Sum**	***p*-Value** **(McNemar)**
IAT ≥ 50	21	14	35	*p* = 0.00098
IAT < 50	1	68	69	
Sum	22	82	104	
(**B**) s-IAT (CO35) and IAT (CO70)				
	**s-IAT ≥ 35**	**s-IAT < 35**	**Sum**	***p*-Value** **(McNemar)**
IAT ≥ 70	2	0	2	*p* < 0.00001
IAT < 70	20	82	102	
Sum	22	82	104	

IAT: Internet Addiction Test; s-IAT: the short version of the Internet Addiction Test, CO: cut-off points.

## Data Availability

The data that support the reported results of this study are available upon request from the corresponding author, M.T.

## Supplementary Materials

The following supporting information can be downloaded at: https://www.mdpi.com/article/10.3390/ijerph20054670/s1, Table S1: Detailed results of this study.
