# Supplementary material for: Clinical Usefulness of a Short Version of the Internet Addiction Test to Screen for Probable Internet Addiction in Adolescents with Autism Spectrum Disorder
Source: Int J Environ Res Public Health. 2023 Mar 6;20(5):4670. doi: 10.3390/ijerph20054670 (PMC10001643; doi:10.3390/ijerph20054670)
Supplement: Supplementary file 1 [file ijerph-20-04670-s001.zip › ijerph-2245984-supplementary.pdf]

Supplemental material

Table S1. Detailed results of this study.

No	Age	Gender	IA status	IAT	s-IAT
1	16	F	Addicted	73	45
2	14	M	Addicted	71	48
3	12	M	Addicted	68	46
4	14	M	Addicted	65	39
5	13	M	Addicted	65	38
6	14	M	Addicted	62	35
7	15	F	Addicted	61	35
8	17	M	Addicted	60	39
9	12	M	Addicted	59	41
10	18	M	Addicted	54	35
11	14	F	Addicted	47	28
12	14	F	Addicted	44	29
13	13	M	Addicted	38	23
14	11	F	Addicted	28	16
15	14	F	Non-addicted	64	40
16	11	F	Non-addicted	63	40
17	15	M	Non-addicted	60	37
18	10	M	Non-addicted	60	33
19	17	F	Non-addicted	59	37
20	13	M	Non-addicted	59	37
21	12	M	Non-addicted	58	34
22	16	M	Non-addicted	58	34
23	11	M	Non-addicted	57	36
24	13	M	Non-addicted	56	36
25	11	M	Non-addicted	56	35
26	18	M	Non-addicted	56	33
27	18	M	Non-addicted	56	32
28	13	M	Non-addicted	54	38
29	15	M	Non-addicted	54	31
30	18	M	Non-addicted	53	35
31	11	M	Non-addicted	53	34
32	17	M	Non-addicted	53	33
33	14	M	Non-addicted	53	29
34	17	M	Non-addicted	51	32
35	18	M	Non-addicted	51	30

36	14	F	Non-addicted	51	30
37	17	M	Non-addicted	50	35
38	14	M	Non-addicted	50	31
39	12	M	Non-addicted	50	30
40	15	M	Non-addicted	49	31
41	18	F	Non-addicted	49	29
42	17	F	Non-addicted	49	28
43	17	F	Non-addicted	49	27
44	17	M	Non-addicted	48	30
45	11	M	Non-addicted	48	24
46	16	M	Non-addicted	47	36
47	13	M	Non-addicted	47	27
48	14	M	Non-addicted	46	33
49	11	M	Non-addicted	46	30
50	13	F	Non-addicted	46	29
51	10	M	Non-addicted	45	32
52	18	M	Non-addicted	45	28
53	11	M	Non-addicted	45	25
54	12	M	Non-addicted	44	33
55	15	M	Non-addicted	44	28
56	18	F	Non-addicted	43	26
57	13	M	Non-addicted	42	28
58	12	M	Non-addicted	42	25
59	12	M	Non-addicted	41	27
60	18	M	Non-addicted	41	24
61	14	M	Non-addicted	40	27
62	16	M	Non-addicted	40	24
63	14	F	Non-addicted	39	29
64	13	F	Non-addicted	39	29
65	13	M	Non-addicted	39	25
66	18	M	Non-addicted	39	22
67	11	M	Non-addicted	38	28
68	16	M	Non-addicted	38	27
69	12	M	Non-addicted	38	25
70	12	M	Non-addicted	38	23
71	15	M	Non-addicted	37	26
72	13	M	Non-addicted	37	24
73	10	M	Non-addicted	36	22
74	18	M	Non-addicted	36	22
75	15	M	Non-addicted	35	18
76	11	M	Non-addicted	33	25

77	13	F	Non-addicted	33	24
78	18	M	Non-addicted	33	20
79	12	M	Non-addicted	33	19
80	18	M	Non-addicted	33	19
81	14	M	Non-addicted	31	21
82	15	M	Non-addicted	31	21
83	11	M	Non-addicted	31	21
84	15	M	Non-addicted	31	20
85	15	F	Non-addicted	31	18
86	14	F	Non-addicted	31	15
87	17	M	Non-addicted	30	20
88	12	F	Non-addicted	30	20
89	12	M	Non-addicted	30	19
90	15	M	Non-addicted	30	17
91	16	F	Non-addicted	28	19
92	16	M	Non-addicted	28	18
93	16	M	Non-addicted	28	18
94	14	M	Non-addicted	28	17
95	15	M	Non-addicted	28	17
96	18	F	Non-addicted	26	18
97	14	M	Non-addicted	26	17
98	14	M	Non-addicted	26	16
99	18	M	Non-addicted	26	14
100	17	M	Non-addicted	25	17
101	10	F	Non-addicted	25	15
102	18	M	Non-addicted	24	16
103	14	F	Non-addicted	24	16
104	17	M	Non-addicted	24	14

M: Male, F: Female, IA: Internet addiction, IAT: Internet Addiction Test, s-IAT: The short version of Internet Addiction Test.
